# Sleep, mood disorders, and the ketogenic diet: potential therapeutic targets for bipolar disorder and schizophrenia

**DOI:** 10.3389/fpsyt.2024.1358578

**Published:** 2024-02-14

**Authors:** Jinyoung Choi, Jiseung Kang, Tae Kim, Christa J. Nehs

**Affiliations:** ^1^ Department of Anesthesia, Critical Care, and Pain Medicine, Massachusetts General Hospital, Harvard Medical School, Boston, MA, United States; ^2^ Division of Sleep Medicine, Harvard Medical School, Boston, MA, United States; ^3^ Department of Biomedical Science and Engineering, Gwangju Institute of Science and Technology, Gwangju, Republic of Korea

**Keywords:** sleep, ketogenic diet, metabolism, bipolar disorder, schizophrenia

## Abstract

Bipolar disorder and schizophrenia are serious psychiatric conditions that cause a significant reduction in quality of life and shortened life expectancy. Treatments including medications and psychosocial support exist, but many people with these disorders still struggle to participate in society and some are resistant to current therapies. Although the exact pathophysiology of bipolar disorder and schizophrenia remains unclear, increasing evidence supports the role of oxidative stress and redox dysregulation as underlying mechanisms. Oxidative stress is an imbalance between the production of reactive oxygen species generated by metabolic processes and antioxidant systems that can cause damage to lipids, proteins, and DNA. Sleep is a critical regulator of metabolic homeostasis and oxidative stress. Disruption of sleep and circadian rhythms contribute to the onset and progression of bipolar disorder and schizophrenia and these disorders often coexist with sleep disorders. Furthermore, sleep deprivation has been associated with increased oxidative stress and worsening mood symptoms. Dysfunctional brain metabolism can be improved by fatty acid derived ketones as the brain readily uses both ketones and glucose as fuel. Ketones have been helpful in many neurological disorders including epilepsy and Alzheimer’s disease. Recent clinical trials using the ketogenic diet suggest positive improvement in symptoms for bipolar disorder and schizophrenia as well. The improvement in psychiatric symptoms from the ketogenic diet is thought to be linked, in part, to restoration of mitochondrial function. These findings encourage further randomized controlled clinical trials, as well as biochemical and mechanistic investigation into the role of metabolism and sleep in psychiatric disorders. This narrative review seeks to clarify the intricate relationship between brain metabolism, sleep, and psychiatric disorders. The review will delve into the initial promising effects of the ketogenic diet on mood stability, examining evidence from both human and animal models of bipolar disorder and schizophrenia. The article concludes with a summary of the current state of affairs and encouragement for future research focused on the role of metabolism and sleep in mood disorders.

## 1 Introduction

Bipolar disorder is characterized by cycles of depression and mania with increased risky behavior. Bipolar disorder affects up to 5% of the population and is the 6th leading cause of disability (1). Schizophrenia is a psychiatric condition characterized by delusions, disorganized speech, hallucinations, and impaired executive functioning that affects up to 1% of the population (2). People with schizophrenia have a 10-20 year shortened life expectancy. Although treatment options, such as medications and psychosocial support are available, many patients continue to grapple with social integration challenges, and some exhibit resistance to existing therapies (3). While the precise pathophysiology of bipolar disorder and schizophrenia remains elusive, a growing body of evidence underscores the pivotal role of oxidative stress and redox dysregulation as an underlying mechanism in bipolar disorder and schizophrenia (4–7). Meanwhile, ketones are neuroprotective including anticonvulsant properties in epilepsy (8–10), reduced oxidative stress and inflammation (11–13), and epigenetic upregulation of neurotrophic factors which could mediate improved mood symptoms (14). Recent pilot clinical trials have indicated the potential benefits of ketone-based interventions for individuals with bipolar disorder and schizophrenia (15–21). However, there remains a noticeable gap in our understanding of the biological mechanisms underlying the positive effects of ketones on psychiatric symptoms. Sleep disorders and disruption in circadian rhythms are recognized as fundamental contributors to the onset and progression of bipolar disorder and schizophrenia (22–24). Research has brought to light evidence of astroglia dysfunction in conditions surpassing antioxidant capacity (25–27), potentially resulting in disruption of circadian rhythms (28, 29). Interestingly, reports indicate neuroglial abnormalities, including a reduction in the overall number of neuroglial cells may provide important clues to the pathogenesis in psychiatric disorders (30, 31). Despite numerous hypotheses and evidence from various disciplines, a multidisciplinary approach to understanding the pathology of schizophrenia and bipolar disorder in relation to sleep abnormalities has yet to be established. In this narrative review, we will provide an overview of the metabolic pathology associated with bipolar disorder and schizophrenia. Subsequently, we will provide evidence of the therapeutic effects of ketogenic diets on psychiatric disorders from preclinical and clinical research. We will also review the changes in metabolism, sleep disorders, and circadian rhythms on psychiatric disorders. The objective of this review is to connect brain metabolism, sleep, and psychiatric disorders.

## 2 Dysfunctional metabolism in bipolar disorder and schizophrenia

In contemporary metabolomics, emerging insights into bipolar disorder reveal notable pathologies, encompassing mitochondrial dysfunction, perturbed energy synthesis, and abnormal mitochondrial morphology (6, 32–35). Mitochondria, the primary contributors to adenosine triphosphate (ATP) synthesis, play a pivotal role in reactive oxygen species generation, apoptosis, and calcium homeostasis (36–39). Among individuals with bipolar disorder, mitochondrial dysfunction leads to diminished energy production (6, 40). This mitochondrial dysfunction coincides with heightened apoptosis, increased reactive oxygen species, oxidative damage, hyperexcitability (41, 42), and a demonstrated elevation in proinflammatory cytokine levels associated with bipolar disorder (41, 43). Beyond mitochondrial dysfunction, structural aberrations in mitochondria have been documented in bipolar disorder patients, manifesting in anomalous mitochondrial structure in the prefrontal cortex, fibroblasts, and lymphocytes (34). Scaini et al. have proposed that an imbalance in mitochondrial fission and fusion processes result in an excess of damaged mitochondria, ultimately contributing to apoptosis (44). Calcium homeostasis, a critical determinant of apoptosis, is regulated by mitochondria through the modulation of intracellular calcium ion concentrations across the mitochondrial membrane. This regulation governs energy production rates, apoptosis, and neuronal excitability (45–47). Notably, bipolar disorder patients exhibit elevated intracellular calcium ion levels during both manic and depressive phases, indicating a connection between bipolar disorder pathophysiology and calcium signaling (48). Furthermore, calcium homeostasis holds significance for neuronal excitability, a crucial element in synaptic plasticity and maintaining excitatory/inhibitory balance (49). Consequently, dysregulation of calcium plays a pivotal role in the pathophysiology of bipolar disorder (50). Bipolar disorder patients also present high lactate levels and reduced intracellular pH in the brain suggesting ATP generation relies on glycolytic metabolism (51, 52). The disruption in the ATP production pathway has been postulated as a potential contributor to the pathogenesis of bipolar disorder (53). Normal ATP levels hinge on both oxidative phosphorylation and glycolysis, implying that a decrease in Na^+^/K^+^-ATPase function may impede oxidative phosphorylation (54). Altered ATP levels can impact neurotransmitter release duration, influencing a neuron’s transition into excitatory or refractory states. Thus, changes in the activation threshold of neurons could contribute to the observed manic and depressed states in bipolar disorder (55).

Schizophrenia is also rooted in dysfunctional cerebral bioenergetics, arising from disruptions in brain cell function, neuroplasticity, and brain circuits, often associated with impaired energy metabolism (56–58). Recent studies have revealed consistent trends in metabolic dysfunction in schizophrenia, including compromised insulin signaling, impaired glucose metabolism (59–62), and dysfunctional astrocyte-neuron coupling leading to impaired lactate shuttling and glycolysis (63–67). These findings highlight a fundamental disruption in key metabolic cycles, notably the tricarboxylic acid (TCA) cycle and oxidative phosphorylation (56). Oxidative phosphorylation, a primary contributor to ATP synthesis, is important for cellular signaling and neuronal activity. When it is disrupted, it can lead to an energy imbalance in the central nervous system, resulting in neuronal dysfunction (68). Moreover, alterations in neurotransmitter systems are evident in schizophrenia patients, characterized by low serotonin, dopamine, and GABA levels in the prefrontal cortex (69–71). Hypofunction of inhibitory GABAergic interneurons is implicated in an imbalance between inhibitory and excitatory processes, a key aspect of schizophrenia pathophysiology (72, 73). In bipolar disorder, reports indicate imbalances in the monoaminergic neurotransmitter system, decreased GABAergic transmission, increased glutamate levels, and heightened NMDA receptor activity (74–77). Furthermore, shared changes in both schizophrenia and bipolar disorder, such as increased lactate and decreased intracellular pH, suggest that glycolysis-dependent bioenergetics may contribute to the risk of metabolic syndrome in psychiatric disorders (78, 79).

Emerging research provides growing evidence supporting oxidative stress as an underlying mechanism in bipolar disorder (4, 5). Reactive oxidative species, natural byproducts of energy metabolism and cellular function, can lead to oxidative stress and cellular damage if not properly eliminated (80). This oxidative stress can also result in mitochondrial dysfunction, another potential factor in bipolar disorder (5, 6). Redox homeostasis is crucial not only for containing tissue damage over time but also for maintaining appropriate signaling in specific biochemical pathways (81). Redox dysregulation is also notably regarded as a pivotal environmental risk factor in the neurodevelopmental context of schizophrenia (7). The balance between cortical excitatory and inhibitory activity, mediated by parvalbumin interneurons (PVI) GABAergic circuits, plays a crucial role in high-frequency neuronal synchrony (82) and is fundamental for normal cognitive, emotional, and social behaviors (83). Alterations in PVI circuits are distinctive features of schizophrenia (84, 85) and have also been identified in bipolar disorder (86). Fast-spiking PVI neurons, rely on heightened metabolic activity and oxidative phosphorylation to support high-frequency discharge (87), exhibit heightened vulnerability to redox dysregulation. Mounting evidence suggests the involvement of mitochondrial dysfunction and augmented reactive oxygen species production in the pathophysiology of schizophrenia (88–90). Prolonged oxidative stress in PVI cells may lead to delays and extensions in the critical period of cortical plasticity, ultimately culminating in the failure to stabilize cortical circuits (91), coupled with inflammatory processes marked by elevated cytokine levels that contribute to neurodegeneration and apoptosis (88, 90, 92).

The hypotheses of bioenergetic dysfunction and redox dysregulation offer a common framework for understanding the pathogenesis of bipolar disorder and schizophrenia. Therefore, future studies might aim to target these metabolic mechanisms to investigate the underlying pathology of mood disorders.

## 3 Similarities between psychiatric disorders and epilepsy

Intriguingly, common underlying pathophysiology exists between epilepsy and bipolar disorder, with biochemical, structural, and functional abnormalities found in primary bipolar disorder potentially occurring secondarily to seizure disorders, both of which are treated with anticonvulsants (93). Ketogenic diet metabolic therapy, which promotes a metabolic state of nutritional ketosis by low-carbohydrate and high-fat intake, has long been established as an adjunctive treatment for epilepsy. The ketogenic diet effectively reduces seizure frequency and severity in drug-resistant epilepsy across pediatric and adult populations (8, 9). Although its exact mechanisms remain incompletely understood, the ketogenic diet likely induces metabolic changes, alterations in neurotransmitter activity, and microbiome modifications contributing to its antiepileptic effects (10, 12, 94). Since its introduction in the 1920s, a substantial body of research, including randomized controlled trials, has supported its efficacy in reducing seizure frequency and enhancing cognitive and behavioral outcomes for those affected by this neurological disorder (95).

The intricate relationship between epilepsy and bipolar disorder can be elucidated through the kindling model, first described by Goddard et al. (96), which provides a framework for understanding the episodic and progressive nature of both disorders (93). Studies have shown that neurobiological alterations, such as changes in second-messenger systems and ion channel functions, are present in both epilepsy and bipolar disorder, reinforcing the hypothesis of a shared pathophysiology (97, 98). These commonalities are further substantiated by the efficacy of antiepileptic drugs in the treatment of both conditions (99). Research into the kindling paradigm has indicated that psychosocial stressors may have more profound effects early in the course of bipolar disorder, with subsequent episodes increasing in frequency and severity—a pattern that aligns with the progression observed in epilepsy (100). The potential for this kindling-like phenomenon in bipolar disorder suggests that long-term prophylaxis may be critical for preventing relapses and mitigating disease progression (98). Moreover, the use of antiepileptic drugs has been reviewed extensively, revealing their significant impact on mood disorders comorbid with epilepsy, highlighting the therapeutic crossover between these disorders (101).

Additionally, epidemiological studies have found a high prevalence of bipolar symptoms among patients with epilepsy, suggesting that the management of bipolar disorder symptoms could be integral to epilepsy treatment strategies (97). Furthermore, neuroimaging studies, such as voxel-based analyses, have uncovered structural brain changes in bipolar disorder that bear resemblance to those found in epilepsy, adding to the body of evidence of shared neurobiological underpinnings (102). This converging evidence emphasizes the necessity for an integrative approach to understanding and treating these complex disorders, underlining the importance of future research in identifying the precise mechanisms that may be responsible for their comorbidity and guiding the development of targeted therapies.

## 4 Effect of the ketogenic diet on psychiatric disorders

Emerging evidence suggests potential positive effects of the ketogenic diet on bipolar disorder and schizophrenia. Case reports document significant improvements in bipolar disorder symptoms, including mood stabilization and decreased anxiety, following ketogenic interventions (15–17). Patients with schizoaffective disorder also have experienced mood and psychotic symptom improvements within a month or achieving remission of psychotic symptoms upon initiating the ketogenic diet (19, 20). Additionally, a cohort study reports symptom improvements and reduced psychotropic medication dosages in patients with bipolar disorder, major depressive disorder, and schizophrenia adhering to a ketogenic diet (18). Another recent cohort study conducted by Needham et al. (21) demonstrated feasibility of the ketogenic diet intervention in patients with bipolar disorder. The authors meticulously documented evidence of ketosis through daily ketone level logs. Notably, their approach included considerations of health economics. This survey encompassed economic expenditure levels, quality of life, and productivity measures among participants during and post the intervention (103). Overall, these clinical studies collectively indicate possible beneficial effects of the ketogenic diet in treating mood disorders and schizophrenia. In our comprehensive analysis (**Supplementary Table 1**), we found that more consistent information on dietary intervention parameters (e.g., dietary ratio of fat and carbohydrate), intervention duration, and tracking of ketosis data is needed for achieving reproducible outcomes in dietary intervention studies. To address these gaps, recent clinical trials that aim to deliver a comprehensive systematic report encompassing metabolite measurement, symptom assessment, and diet adherence continuity are in progress across multiple centers (104). Meanwhile, conducting more randomized controlled trials with ketogenic diet interventions is imperative to establish efficacy, which patient populations benefit the most, and level of ketosis needed.

The ketogenic diet works through multiple pathways which may contribute to its effectiveness for multiple neurological conditions. These include increasing available fuel/ATP, decreasing oxidative stress, decreasing inflammation, direct signaling through HCARs, epigenetic regulation through HDAC inhibition, microbiome changes, altering neurotransmitters such as glutamate and GABA balance, improve mitochondrial function (105). Here we describe some of these mechanisms. Improvements in symptoms of mental disorders with the ketogenic diet may be ascribed to the circumvention or restoration of mitochondrial function through alternative energy metabolism via ketosis (106, 107). Both animal models and human subjects have illustrated heightened mitochondrial biogenesis, mass, and energy production following ketogenic diet treatment (108, 109). Rats achieving chronic ketosis displayed elevated mitochondrial proteins and genes associated with oxidative phosphorylation, conferring increased resilience to metabolic stress compared to control rats (110). Additionally, the ketogenic diet can modulate neurotransmitter balance and release by enhancing GABA biosynthesis and glutamate metabolism, potentially contributing to rebalancing disturbed GABA concentrations that influence the symptomatology of psychiatric diseases (111, 112). A recent preclinical study using an acute NMDA receptor hypofunction model of schizophrenia demonstrated that feeding C57BL/6 mice a low carbohydrate/high-fat ketogenic diet for seven weeks prevented behavioral abnormalities induced by pharmacological inhibition of NMDA glutamate receptors (113). Furthermore, ketosis is hypothesized to modify ion concentrations, both extracellular and intracellular, mirroring therapeutic effects observed with mood stabilizers (14). Indeed, rats provided ketone supplements, including medium-chain triglycerides which can be converted into ketones, ameliorated anxiety- and depression-related behaviors, suggesting that ketone supplementation may represent a promising anxiolytic strategy through a novel means of inducing ketosis (114, 115).

Mitochondrial dysfunction, oxidative stress, and microglial activation have been implicated in the pathophysiology of schizophrenia (88–90, 116). Elevated ketone body levels were reported in schizophrenic patients, demonstrating a positive correlation between changes of executive function and the level of β-hydroxybutyrate, indicating a potential need for supplementary energy supply through ketone bodies in schizoaffective patients (117). Furthermore, ketones decreased neuroinflammation caused by oxidative stress or mitochondrial dysfunction via neuroprotective effects (118–120). *In vitro* studies have shown that a ketogenic diet exerts essential neuroprotective impacts by reducing the production of the proinflammatory cytokine interleukin (IL)-17 and increasing the levels of the anti-inflammatory cytokine IL-10 (121). These neuroprotective effects may be mediated by the regulation of immune cell response, suppressing inflammasome-related microglial inflammation progression (122). These effects were associated with reduced amounts of IL-1β and decreased release of reactive oxygen species (123). Ketones also block NLRP3 inflammasome mediated inflammation (120). *In vivo* studies revealed that β-hydroxybutyrate suppressed IL-6 and TNF-α production, induced brain-derived neurotrophic factor, and suppressed microglial progress retraction, along with depression-like behaviors (117). Therefore, the neurotrophic and antioxidant effects of ketone bodies could potentially offer benefits for bipolar disorder depression, which is associated with downregulated gene expression of antioxidant enzymes and increased oxidative damage (124–126). Meanwhile, the potential antidepressant effect of the ketogenic diet may involve the regulation of G-Protein Coupled Receptors (GPCRs) signaling, which transmits extracellular signals into the cell. Epigenetic effects on GPCRs could contribute to biochemical changes in psychiatric disorders (127). Environmental factors (e.g., chronic or acute stress) influence genetic risk, and studies associate candidate genes encoding GPCRs with schizophrenia (128). Notably, β-hydroxybutyrate reduces inflammation by engaging GPCRs, particularly GPR109A or Hydroxycarboxylic Acid Receptor 2 (HCAR_2_) (129). Studies indicate that β-hydroxybutyrate binding to GPR109A reduces lipid metabolism and inflammation, supported by animal studies. One study demonstrated that β-hydroxybutyrate inhibits the production of pro-inflammatory enzymes, IL-1β, IL-6, and TNF-α, from microglia, mediated by GPR109A (130). These findings suggest that ketone bodies can modulate various pathways related to inflammation, a significant factor in psychiatric disorders.

Although the exact pathophysiology of bipolar disorder and schizophrenia remains unclear, evidence supports the notion that impaired energy metabolism and related oxidative stress plays a crucial role in symptom manifestation. The current evidence from clinical trials of the ketogenic diet’s antipsychotic effects on bipolar disorder and schizophrenia is promising. However, more clinical trials are needed to establish efficacy, which patient populations it benefits the most, and the level of ketosis needed to achieve symptom remission. Such studies are difficult given the complex nature of executing large well controlled dietary clinical trials and a team of psychiatrists and knowledgeable nutritionists are needed. Patient education and continuous monitoring for adverse events are required. Patients with kidney failure or who are prone to kidney stones should be monitored closely if they choose to follow a ketogenic diet as high protein intake can increase the risk of kidney stones. Making sure to stay hydrated and supplementing with potassium citrate can mitigate the risk of developing kidney stones. Patients with psychiatric disorders often have accompanying metabolic disorders like diabetes and the fact that these two conditions often coexist highlights the connection between them. In fact, large multiyear studies of patients with type 2 diabetes have shown that the ketogenic diet can reverse all symptoms of diabetes and patients are able to deprescribe most of their diabetes medications (131) so ketogenic diets are safe in diabetics. Taken together, both randomized clinical trials and preclinical research focusing on cellular and molecular mechanisms of the ketogenic diet’s effects on psychiatric disorders are needed. Translational research using animal models is a promising additional approach to validate and elucidate the metabolic mechanisms underlying antipsychotic effects of a ketogenic diet in mood disorders and schizoaffective diseases. See **Supplementary Table 1** for a rapid systematic review of clinical studies looking at ketogenic diet interventions in psychiatric diseases.

## 5 Sleep disorders and disruption of circadian rhythms in bipolar disorder and schizophrenia

Patients with bipolar disorder frequently experience hypersomnia (132, 133), and disruption of circadian rhythms, which are thought to be fundamental contributors to bipolar disorder onset and progression (22, 23). Sleep abnormalities in bipolar disorder vary between acute depressive and manic episodes (134). During depressive episodes, patients generally have increased total sleep time and time in bed but lower sleep efficiency, while manic episodes are characterized by reduced sleep time and increased rapid eye movement (REM) sleep, also with lower sleep efficiency (135). Bipolar disorder typically involves prolonged sleep onset latency, increased frequency of rapid eye movement sleep across all stages (136), and irregular sleep patterns that may increase the risk of recurrence of manic or depressive episodes (137–139).

In schizophrenia, about half of the patients exhibit significant disruption of circadian rhythms (24), characterized by increased sleep onset latency, decreased total sleep time, and reduced sleep efficiency (140). Schizophrenia affects various neurotransmitter systems involved and overlaps with those in sleep regulation (141). For example, one of the hypotheses of neural mechanisms of psychiatric symptoms of schizophrenia is aberrant dopamine signaling, which is one of the major regulators of sleep and wakefulness (142). Patients with schizophrenia also have a significant reduction in REM sleep latency and sleep density (143, 144) and insomnia is associated with psychopathology of schizophrenia (145). Additionally, spindle deficits in schizophrenia, particularly in the frontal-parietal and prefrontal areas, are observed in patients on antipsychotics (146–148) and in patients with first-episode psychosis who are antipsychotic-naïve or minimally treated (149). Patients with first-episode psychosis also show reduced slow wave density in frontal-central regions, including the prefrontal cortex (150). In schizophrenia, spindle density is correlated with positive symptom intensity, working memory deficits, and the severity of negative symptoms in patients with first-episode psychosis (147, 149, 151). These abnormalities are linked to reduced mediodorsal thalamic volumes (148) and decreased connectivity between the mediodorsal thalamus and prefrontal cortex, evident in fMRI studies (152). Altered resting-state connectivity in the thalamocortical network is also negatively associated with spindle density (153).

We also found decreased total sleep time, lower sleep efficiency, and longer sleep latency in schizophrenia from multiple meta-analyses (**Supplementary Table 2**). In addition to the sleep disruptions, another significant sleep disorder commonly found in both schizophrenia and bipolar disorder is obstructive sleep apnea (154). Obstructive sleep apnea is characterized by repeated episodes of partial or complete obstruction of the upper airway during sleep, leading to disrupted sleep-wake cycles and decreased oxygen saturation (154). This disorder is particularly related to metabolism, as it often co-occurs with metabolic syndrome, involving increased blood pressure, high blood sugar, more body fat and waist circumference, and low high density lipoprotein cholesterol or high triglycerides (155). Patients with psychiatric disorders, including schizophrenia and bipolar disorder, show a higher prevalence of metabolic syndrome (141, 156), suggesting a potential common metabolic pathway that may contribute to the development and exacerbation of both psychiatric and sleep disorders.

Bipolar disorder and major depression also show widespread glial abnormalities (157–159). In the suprachiasmatic nucleus, the master circadian clock, astrocytes regulate circadian rhythms via glial fibrillary acidic protein expression, influencing glutamate levels (28). Additionally, accumulated data suggests that, under physiological conditions, astrocytes play a crucial role as the primary source of adenosine (29, 160, 161). Sleep homeostasis is regulated by the accumulation of adenosine in the brain during wakefulness and its subsequent decline during sleep (161–163). Interestingly, postmortem studies of patients with major depressive disorder and bipolar disorder reveal a decreased number of glial cells (30). It implies that decreased number of glial cells in the suprachiasmatic nucleus can contribute to the disruption of circadian rhythms in patients with bipolar disorder (160, 164). In addition, chronic stress causes astrocyte structural atrophy and loss of function, decreasing astrocyte support of neural transmission, leading to depressive behavior (165). This astrocyte asthenia in mood disorders may contribute to impaired sleep regulation, linking immune dysfunction as a potential mediator of the bidirectional relationship between sleep dysfunction and bipolar disorder (166, 167).

Despite numerous hypotheses and evidence from various disciplines, a multidisciplinary approach to understanding the pathology of schizophrenia and bipolar disorder in relation to sleep abnormalities has yet to be established. Therefore, further research is needed for a comprehensive understanding of the underlying mechanisms and relationship between circadian abnormalities and the pathophysiology of mood disorders, including bipolar disorder and schizophrenia. See **Supplementary Table 2** for a rapid systematic review of meta-analyses looking at sleep disturbances and their associations with psychiatric diseases.

## 6 Effect of the ketogenic diet on sleep and circadian rhythms

It has long been known that sleep and metabolism are related. For example, sleep deprivation alters glucose metabolism (168) and altered glycemic control in diabetics correlates with sleep quality (169, 170). In fact, the level of carbohydrate intake is correlated with sleep quality, where more carbohydrates lead to lower subjective sleep quality (171), less slow wave sleep, and more REM sleep (172, 173). The role of ketones in modulating sleep [reviewed in (174, 175)] has support from both rodent and human studies. Intracerebral ventricular injection of the ketone body, acetoacetate, in mice increases delta power during sleep (176). The ketogenic diet in both diabetics (177) and psychiatric (178) patients improved their subjective sleep quality measured by the Pittsburgh Sleep Quality Index. Narcoleptic patients on a ketogenic diet showed reduced narcolepsy symptoms (179). The ketogenic diet has also been shown to improve migraine patients sleep quality and decrease insomnia (180). Women with obesity on a very low carbohydrate diet for 31 days showed improved sleep quality that correlated with the change in fat mass (181). Children with epilepsy showed improved sleep quality, normalized sleep architecture with increased REM sleep, and decreased daytime sleep (182).

The ketogenic diet is becoming more widely used in weight loss and medical interventions such as diabetes and psychiatry but there are few high-quality long-term studies investigating its effect on objective sleep criteria. Consistent with the idea that ketones improve sleep quality, a study measuring sleep across 4 days of fasting, where ketones are known to rise, found NREM sleep increased and REM sleep decreased (183). Exogenous ketone ester supplements are able to counter intense exercise induced decrease in REM sleep and improve sleep efficiency (184). When ketogenic diets are tested in young healthy people, there have been varying results. Short-term ketogenic diet consumption increased slow wave sleep and reduced REM sleep in one study (185) whereas 3 weeks of the ketogenic diet did not improve patients sleep further in young healthy people (186). These differences may be due to young healthy people already having high quality sleep so there is not as much room for improvement. In addition, keto-adaptation can take longer than 3 weeks to occur (187) so longer interventions in more people are needed.

Ketone bodies also function as metabolic and signaling mediators impacting circadian rhythms. The composition of food and meal timing can influence circadian activity, and ketone bodies, along with nutritional challenges from a ketogenic diet, can modulate diurnal rhythms in peripheral tissues, interpreted differently by tissue-specific clocks (188–193). Additionally, ketogenic diet feeding induces circadian transcriptional reprogramming of intestinal energy metabolism, controlled by core clock-independent mechanisms (190).

While disrupted sleep and circadian rhythms are fundamental contributors to the onset and progression of bipolar disorder (22–24), understanding the relationship between sleep and mood disorders is incomplete. In schizophrenia research, macrostructural changes in sleep are evident, and sleep oscillations (e.g., sleep spindle and slow oscillations) are affected by schizoaffective disorder. However, the role of sleep in understanding the mechanism of schizophrenia is often overlooked (194). Therefore, further research should prioritize identifying specific sleep parameters as reliable indicators for predicting mood disorder progression and evaluating the effectiveness of therapeutic interventions for mental diseases.

## 7 Conclusions: sleep, metabolism, and mental health – current state and future directions

This review has comprehensively assessed the multiple theories underlying the pathology of bipolar disorder and schizophrenia and explored potential ketosis therapeutic mechanisms. Shared pathophysiological aspects between bipolar disorder and schizophrenia include suspected associations with bioenergetic dysfunction, potentially stemming from mitochondrial dysfunction, alterations in the TCA cycle, and disturbances in neurotransmitter systems (6, 32–35, 56–58). Among these factors, mitochondrial dysfunction has downstream effects, contributing to disruption in energy supply, increased oxidative stress, apoptosis, and imbalances in calcium ion concentrations, with interplay among these effects (44–47). Moreover, prolonged oxidative stress in the circuitry of neurotransmitters, e.g., parvalbumin interneurons in GABAergic circuits, have been identified in schizophrenia and bipolar disorder (84–86). This implies that redox dysregulation can be regarded as a notable environmental risk factor for neuronal dysfunction in psychiatric diseases which may relate to psychotic symptoms (5, 7, 81).

The therapeutic potential of the ketogenic diet for bipolar disorder and schizophrenia was also discussed. Recent clinical trials have offered substantial evidence of the therapeutic effects of a ketogenic diet in bipolar disorder and schizophrenia (15–20). Patient commitment is required to adhere to the dietary guidelines to achieve and maintain ketosis. There are well-controlled clinical trials with systematic protocols that are promising for understanding the therapeutic effects of the ketogenic diet in psychiatric patients (21, 104). Nevertheless, the complexity of these effects warrants concurrent preclinical research to elucidate the intricate impact of ketosis on the pathology of bipolar disorder and schizophrenia. Preclinical studies have suggested the potential benefits of ketone bodies on mental health, supporting mitochondrial function and exhibiting improved energy production and gene expression associated with oxidative phosphorylation (118–120). Both human and animal studies also validate the anxiolytic, antioxidant, and neuroprotective effects of ketosis in the context of oxidative stress and inflammation (108–110, 113–115).

We also summarized sleep disorders and disruption in circadian rhythms in mood and psychiatric disorders. Patients with bipolar disorder experience hypersomnia and insomnia depending on the phase of the disease and sleep disturbances have negative impacts on the course of psychiatric illness in both of bipolar disorder and schizophrenia (22, 195, 196). In schizophrenia, prominent deficiencies in sleep spindles, associated with cognitive symptoms, are observed (146–149, 151). Moreover, patients with bipolar disorder and schizophrenia exhibit a higher prevalence of metabolic syndrome, often accompanied by obstructive sleep apnea, indicating a common metabolic pathology (154). Glial abnormalities, particularly in astrocytes, are implicated in the pathology of mood disorders (157–159), with astrocytes being a primary source of adenosine crucial for sleep homeostasis (29, 160, 161). Consequently, damaged or decreased glial cells in the suprachiasmatic nucleus, the master circadian clock, may contribute to impaired sleep regulation (160, 164).

This review has examined the evidence of bioenergy dysfunction and redox dysregulation in the pathogenesis of bipolar disorder and schizophrenia. These foundational pathologies in psychiatric disorders have been substantiated through diverse research approaches, encompassing human postmortem analysis, preclinical studies involving animal models or cell cultures, and molecular science research detailing the favorable impacts of ketone bodies on energy metabolism, redox homeostasis, and neuroprotection. These comprehensive insights advocate for the necessity of translational research exploring the ketogenic diet as a therapeutic approach for mood disorders and psychiatric diseases, potentially supporting ongoing clinical trials.

Furthermore, the critical role of sleep in bipolar disorder and schizophrenia research has been underscored. While sleep-related observations for biomarker research are currently undervalued, our emphasis on the impact of glial abnormalities in sleep highlights the need for future preclinical research to unravel the metabolic mechanisms underpinning sleep disorders in mental health. Oxidative stress emerges as a primary pathology leading to downstream damage in the neural-glial network of individuals with bipolar disorder and schizophrenia. Considering the neuroprotective effects of ketone bodies and their capacity to mitigate oxidative stress, investigating the ketogenic diet in sleep research becomes a rational approach to elucidate the beneficial effects of ketosis on sleep disorders.

This comprehensive narrative review highlights the intricate relationship between sleep disorders, dysfunctional metabolism, and mood disorders, as they share similar pathologies and therapeutic targets—specifically, oxidative stress and the ketogenic diet. Despite a substantial body of evidence on each research topic, there is a noticeable dearth of studies considering these interconnected pathological mechanisms in bipolar disorder and schizophrenia. Therefore, we propose that future research in mood and schizoaffective disorders, in the form of both randomized controlled clinical trials and translational studies in animal models, should adopt a multidisciplinary approach to explore the interplay between sleep, metabolism, and mood disorders. This approach is poised to uncover the pathology and therapeutic targets of these diseases, and we anticipate that translational research in these domains will usher in a new chapter in psychiatric disorder research.

## Author contributions

JC: Conceptualization, Investigation, Writing – original draft, Writing – review & editing. JK: Investigation, Writing – original draft, Writing – review & editing. TK: Investigation, Writing – original draft, Writing – review & editing. CN: Conceptualization, Investigation, Supervision, Writing – original draft, Writing – review & editing.
