# Supplementary material for: Sleep, mood disorders, and the ketogenic diet: potential therapeutic targets for bipolar disorder and schizophrenia
Source: Front Psychiatry. 2024 Feb 14;15:1358578. doi: 10.3389/fpsyt.2024.1358578 (PMC10899493; doi:10.3389/fpsyt.2024.1358578)
Supplement: Supplementary file 1 [file Table_1.docx]

Supplementary data

| **Author (Year), Study type** | **Number of participants** | | **Dietary ratio, duration** | | **Achieved Ketone level** | | **Effect of Ketogenic diet** | | **Other comments** | |
| --- | --- | --- | --- | --- | --- | --- | --- | --- | --- | --- |
| Needham N (2023),  Cohort Study [1] | 27 patients with bipolar disorder (who had been clinically euthymic for three months) were recruited,  20 completed | | 60-75% fat, 5-7% carbohydrate + additional protein,  6-8 weeks | | 1.3 ± 0.8 mmol/L | | Weight loss (9.9 lb in average)  Daily measurement of clinical outcomes will be reported in a future paper. | | The ketogenic diet is feasible for bipolar disorder patients to follow with high compliance and mild adverse events.  Minor adverse events included fatigue, constipation, drowsiness and hunger.  1 patient experienced euglycemic diabetic ketoacidosis on the third day of the intervention (7.1 mmol/L of ketone levels) likely due to continued use of an SGLT2 inhibitor. Proper dietician counseling is needed as SGLT2 inhibitors are recommended to be discontinued before starting a ketogenic diet. | |
| Chmiel I (2022),  Case Report [2] | One patient with bipolar affective disorder,  Completed | | 80% fat, 15% protein, 5% carbohydrate,  52 weeks (1 year) | | 1.5-3 mmol/L | | Improvement in the patient’s mood, increased energy during the day, progression of cognitive functions, improved sleep quality, improved concentration, and lack of anxiety. Periods of remission were even longer: 2–4 weeks, interrupted by a mild, short depression lasting 1–2 days, without hypomania. The doses of antipsychotic drugs were reduced to 100 mg of lamotrigine and 100 mg of quetiapine per day. There was no significant change in body weight (BMI 22.6–23.4). | | The author suggested that the ketogenic diet may have similarities with antiepileptic drugs | |
| Danan A (2022),  Retro-spective Analysis [3] | 31 patients with severe persistent mental illness (major depressive disorder, bipolar disorder, and schizo-affective disorder),  28 completed | | 75-80% fat, 15-20% protein, and carbohydrate restriction (maximum 20 total grams per day),  8.4 weeks (59.1 (±49.6) days) | | Not provided | | Patients with a primary diagnosis of schizoaffective disorder exhibited improvement in PANSS scores, with the mean (SD) Positive and Negative Syndrome Scale (PANSS) score falling from 91.4 (15.3) to 49.3 (6.9), P<0.001, Cohen’s d=3.5.  Patients who were administered Hamilton Depression Rating Scale (HAM-D) exhibited improvement in HAM-D scores with the mean (SD) HAM-D score falling from 25.4 (6.3) to 7.7 (4.2), P < 0.001, Cohen’s d = 3.1.  Medication requirements (Clinical Global Impressions Scale, CGI-S) improved from 4.9 (1.2) to 2.0 (1.1), P < 0.001, Cohen’s d = 3.8.  Average weight loss from 198.5 (42.1) to 187.7 (41.5) lb and BMI change from 31.9 (6.7) to 30.2 (10.3) kg/m2 were both significant (P < 0.001). | | Patients were already hospitalized allowing for more control of their diet. Key to the successful transition to a ketogenic diet in any population is dietary education and support. In this study, prepared meals and snacks were provided. | |
| Saraga M (2020),  Case Report [4] | One patient with type I bipolar disorder,  Completed | | 66-75% fat, 34-25% protein & carbohydrate  (Duration is not provided) | | 0.28-2.2 mmol/L | | Decrease in anxiety and the maintenance of euthymia in spite of markedly stressful life events leading to progressively less sensitivity to dietary lapses. Subject stopped sertindole. | |  | |
| Cox N (2019),  Case Report [5] | One patient with major depressive disorder and type II diabetes,  Completed | | 65% fat, 25% protein, 10% carbohydrate,  12 weeks | | 1.5 mmol/L | | The Patient Health Questionnaire 9 (PHQ-9) score was substantially decreased from 17 (moderately severe depression) to 0 (minimal depression). The General Self-Efficacy Scale (GSE) also improved from 20 to 39.  The patient self-reported a heightened self-confidence, increased self-efficacy, increased energy, improved sleep and stability in mood with clearer cognition. | | Significant correlations were observed between reduced HgA1c and HOMA-IR, depressive symptoms, generalized self-efficacy and weight via regression analyses (p=0.01-0.04) | |
| Palmer CM (2019),  Case Report [6] | Two patients with schizo-phrenia,  Both completed | | No information about dietary ratio and duration | | Not provided | | Subjects remained free of psychotic symptoms with less antipsychotic medications, had weight loss, and were able to live independently. | | Case studies support the need for more randomized controlled trials to establish the safety and efficacy of the ketogenic diet for psychotic disorders. | |
| Palmer CM (2017),  Case Report [7] | Two patients with schizo-affective disorder with history of major depression,  Both completed | | No information about dietary ratio and duration, but both subjects’ diet consisted of coffee with medium chain triglyceride (MCT) oil and butter, eggs, poultry, vegetables | | Not provided | | Positive and Negative Symptom Scale (PANSS) score change was from 98 to 49 for the first subject and from 107 to 70 for the second subject with the ketogenic diet. First subject lost 104 lb of weight and second subject lost 30 lb. | | The ketogenic diet resulted in a significant decrease in both positive and negative symptoms. The effect was only present during ketosis, and was quickly reversed when the ketogenic diet was stopped. | |
| Phelps JR (2013),  Case Report [8] | Two patients with type II bipolar disorder,  Both completed | | No information about dietary ratio *for one participant*,  for 2 years  *Another participant* maintains a diet about 70% fat, 22% protein and 8% carbohydrate,  for 3 years | | 0.27-4 mmol/L for one subject (for 7 months),  and ketone level of another subject was not provided | | Participants were able to lower and ultimately stop lamotrigine while maintaining as good or better symptom control. | | Monitoring of renal function could be useful during ketogenic diet treatment  Sustainability can be one challenge associated with implementing the ketogenic diet and sustained contact with the medical team is helpful | |
| **Supplemental Table 1.** Rapid systematic review of clinical studies with ketogenic diet intervention in mood disorders and schizophrenia. Studies were collected by searching in PubMed with query ‘(Ketogenic diet) AND ((mental disorder) OR (mood disorder) OR (bipolar disorder) OR (affective disorder) OR (schizophrenia) OR (schizoaffective disorder))’. We also added three more articles that we already found during the initial writing of manuscript (Saraga et al., 2020; Danan et al., 2022; and Needham et al., 2023). The table contains articles which were published between 2010 and 2024. We excluded literature which were not written in English, studies for neurodegenerative diseases (e.g., Parkinson’s or Alzheimer’s) and non-human translational research. Values in round brackets are standard deviations. **Author (Year)** | | **Psychiatric diseases** | | **Sleep disorder or Quantitative measure** | | **Number of studies,**  **Number of subjects** | | **Main findings** | |  |
| Miller BJ (2023) [9] | | Schizophrenia | | Insomnia | | 10 studies,  3,428 patients with schizophrenia | | Insomnia was associated significantly with psychopathology of Schizophrenia (ES 0.16 [0.09 0.23], p < 0.01). | |  |
| Dondé C (2022) [10] | | First episode of psychosis | | Total sleep time, sleep efficiency, sleep latency | | 13 studies,  537 patients with early stages of psychosis, and 360 controls | | Shorter total sleep time (ES -0.44 [-0.67 -0.21]), lower sleep efficiency (ES -0.72 [-1.08 -0.36]), longer sleep onset latency (ES 0.75 [0.45 1.06]), and longer duration of wake after sleep onset (ES 0.49 [0.21 0.77]) were observed in early stages of psychosis compared to controls, no difference in electroencephalographic parameters. | |  |
| Aguiar KR (2021) [11] | | Bipolar disorder (Offspring) | | Daytime sleepiness, sleep duration, sleep efficiency, sleep latency | | 4 studies,  43 offspring of parents with bipolar disorder, 42 offspring of control parents | | Offspring of parents with bipolar disorder had greater daytime sleepiness (ES 0.39 [0.04 0.74], p = 0.03)), but no significant difference in sleep duration (ES -0.03 [-0.36 0.29], p = 0.83), sleep efficiency (ES -0.20 ([-0.49 0.09], p = 0.17)), and sleep latency (ES -0.21 [-0.80 0.39], p = 0.50)). | |  |
| Grigolon RB (2019) [12] | | Bipolar disorder | | Hypersomnia | | 10 studies,  1,824 patients with bipolar disorder | | Overall estimate of prevalence of hypersomnia in bipolar disorder cases was 29.9% ([25.8 34.1], I2 = 59.2; p < .05). | |  |
| Baglioni C (2016) [13] | | Major depressive disorder, anxiety disorders, schizophrenia | | Sleep continuity, sleep depth | | 38 studies with patients with major depressive disorder (n=1,524) and controls (n=1,128), 21 studies with patients with anxiety disorder (n=397) and controls (n=409), and 8 studies with patients with schizophrenia (n=154) and controls (n=121) | | Sleep continuity (related withsleep efficiency, sleep latency, and number of awakenings) was associated with major depressive disorder (ES -0.9 [0.12], p<0.0001), anxiety disorders (ES -0.59 [0.17], p=0.003), and schizophrenia (ES -1.03 [0.12], p<0.0001). | |  |
| Stubbs B (2016) [14] | | Major depressive disorder, bipolar disorder, schizophrenia | | Obstructive sleep apnea | | 4 studies with patients with major depressive disorder (n=378), 5 studies in patients with bipolar disorder (n=681), 3 studies with schizophrenia (n=329) | | Prevalence of obstruct sleep apnea is observed in patients with major depressive disorder 25.2% ([10.2 50.2], p<0.01), bipolar disorder 24.5% ([10.6 47.1], p<0.01), and schizophrenia 15.4% ([5.3 37.1], p<0.01). | |  |
| Geoffroy PA (2015) [15] | | Bipolar disorder (remitted) | | Sleep duration, sleep latency, sleep efficiency, wake time after sleep onset | | 9 studies with patients with remitted bipolar disorder (n=202), and controls (n=210) | | Significant differences in sleep latency (ES 0.51 [0.28-0.73], z=4.43, p<0.00001), sleep duration (ES 0.57 [0.30-0.84], z=4.16, p<0.0001), wake after sleep onset (ES 0.28 [0.06-0.50], z=2.48, p=0.01) and sleep efficiency (ES -0.38 [-0.70-0.07], z=2.4, p=0.02) between controls and patients with remitted bipolar disorder. | |  |
| Chouinard S (2004) [16] | | Schizophrenia | | Total sleep time, sleep efficiency, sleep latency | | 20 studies  321 patients with schizophrenia and 331 controls | | Patients with schizophrenia have longer sleep latency (ES 1.34 [0.44 2.24]), shorter total sleep time (ES -1.50 [-2.47 -0.49]), and lower sleep efficiency (ES -1.58 [-2.41 -0.74]) compared to controls. | |  |
| Benca RM (1992) [17] | | Affective disorder, anxiety disorders, schizophrenia | | Total sleep time, sleep efficiency, sleep latency | | 115 studies with patients with affective disorder (n=3,689), 159 studies with patients with anxiety disorders (n=159), and 12 studies with patients with schizophrenia (n=239) | | Patients with affective disorders, anxiety disorders, and schizophrenia (age range 20-50) showed decreased total sleep time, sleep efficiency and increased sleep latency (p<0.05). | |  |

**Supplemental Table 2.** Rapid systematic review of meta-analysis studies with sleep disorders (or sleep disturbances) and psychiatric diseases. Studies were collected by searching in PubMed with query ‘((sleep disorder) AND (bipolar disorder)) OR ((sleep disorder) AND (schizophrenia))’. The table contains articles which were published between 1963 and 2024. We excluded literature which were not written in English, studies of therapeutic intervention, studies addressing suicidal association only, and genetic meta-analysis studies (e.g., genome-wide association studies). Values in square brackets are standard errors. Abbreviation – ES: effect size.

# References

1. Needham, N., Campbell, I. H., Grossi, H., Kamenska, I., Rigby, B. P., Simpson, S. A., McIntosh, E., Bahuguna, P., Meadowcroft, B., Creasy, F., Mitchell-Grigorjeva, M., Norrie, J., Thompson, G., Gibbs, M. C., McLellan, A., Fisher, C., Moses, T., Burgess, K., Brown, R., . . . Smith, D. J. (2023). Pilot study of a ketogenic diet in bipolar disorder. *BJPsych Open*, *9*(6), e176. <https://doi.org/10.1192/bjo.2023.568>
2. Chmiel, I. (2022). Ketogenic diet in therapy of bipolar affective disorder - case report and literature review. *Psychiatr Pol*, *56*(6), 1345-1363. <https://doi.org/10.12740/PP/OnlineFirst/136356> (Dieta ketogenna w terapii zaburzenia afektywnego dwubiegunowego - opis przypadku i przeglad literatury.)
3. Danan, A., Westman, E. C., Saslow, L. R., & Ede, G. (2022). The Ketogenic Diet for Refractory Mental Illness: A Retrospective Analysis of 31 Inpatients. *Front Psychiatry*, *13*, 951376. <https://doi.org/10.3389/fpsyt.2022.951376>
4. Saraga, M., Misson, N., & Cattani, E. (2020). Ketogenic diet in bipolar disorder. *Bipolar Disord*, *22*(7), 765. <https://doi.org/10.1111/bdi.13013>
5. Cox, N., Gibas, S., Salisbury, M., Gomer, J., & Gibas, K. (2019). Ketogenic diets potentially reverse Type II diabetes and ameliorate clinical depression: A case study. *Diabetes Metab Syndr*, *13*(2), 1475-1479. <https://doi.org/10.1016/j.dsx.2019.01.055>
6. Palmer, C. M., Gilbert-Jaramillo, J., & Westman, E. C. (2019). The ketogenic diet and remission of psychotic symptoms in schizophrenia: Two case studies. *Schizophr Res*, *208*, 439-440. <https://doi.org/10.1016/j.schres.2019.03.019>
7. Palmer, C. M. (2017). Ketogenic diet in the treatment of schizoaffective disorder: Two case studies. *Schizophrenia Research*, *189*, 208-209. <https://doi.org/https://doi.org/10.1016/j.schres.2017.01.053>
8. Phelps, J. R., Siemers, S. V., & El-Mallakh, R. S. (2013). The ketogenic diet for type II bipolar disorder. *Neurocase*, *19*(5), 423-426. <https://doi.org/10.1080/13554794.2012.690421>
9. Miller, B. J., & McCall, W. V. (2023). Meta-analysis of insomnia, suicide, and psychopathology in schizophrenia. *Curr Opin Psychiatry*, *36*(3), 156-165. <https://doi.org/10.1097/YCO.0000000000000856>
10. Donde, C., Jaffiol, A., Khouri, C., Pouchon, A., Tamisier, R., Lejoyeux, M., d'Ortho, M. P., Polosan, M., & Geoffroy, P. A. (2022). Sleep disturbances in early clinical stages of psychotic and bipolar disorders: A meta-analysis. *Aust N Z J Psychiatry*, *56*(9), 1068-1079. <https://doi.org/10.1177/00048674211068395>
11. Aguiar, K. R., Cabelleira, M. D., Montezano, B. B., Jansen, K., & de Azevedo Cardoso, T. (2021). Sleep alterations as a predictor of bipolar disorder among offspring of parents with bipolar disorder: a systematic review and meta-analysis. *Trends Psychiatry Psychother*, *43*(4), 256-269. <https://doi.org/10.47626/2237-6089-2021-0256>
12. Grigolon, R. B., Trevizol, A. P., Cerqueira, R. O., Lee, Y., Mansur, R. B., McIntyre, R. S., & Brietzke, E. (2019). Hypersomnia and Bipolar Disorder: A systematic review and meta-analysis of proportion. *J Affect Disord*, *246*, 659-666. <https://doi.org/10.1016/j.jad.2018.12.030>
13. Baglioni, C., Nanovska, S., Regen, W., Spiegelhalder, K., Feige, B., Nissen, C., Reynolds, C. F., & Riemann, D. (2016). Sleep and mental disorders: A meta-analysis of polysomnographic research. *Psychol Bull*, *142*(9), 969-990. <https://doi.org/10.1037/bul0000053>
14. Stubbs, B., Vancampfort, D., Veronese, N., Solmi, M., Gaughran, F., Manu, P., Rosenbaum, S., De Hert, M., & Fornaro, M. (2016). The prevalence and predictors of obstructive sleep apnea in major depressive disorder, bipolar disorder and schizophrenia: A systematic review and meta-analysis. *J Affect Disord*, *197*, 259-267. <https://doi.org/10.1016/j.jad.2016.02.060>
15. Geoffroy, P. A., Scott, J., Boudebesse, C., Lajnef, M., Henry, C., Leboyer, M., Bellivier, F., & Etain, B. (2015). Sleep in patients with remitted bipolar disorders: a meta-analysis of actigraphy studies. *Acta Psychiatr Scand*, *131*(2), 89-99. <https://doi.org/10.1111/acps.12367>
16. Chouinard, S., Poulin, J., Stip, E., & Godbout, R. (2004). Sleep in untreated patients with schizophrenia: a meta-analysis. *Schizophr Bull*, *30*(4), 957-967. <https://doi.org/10.1093/oxfordjournals.schbul.a007145>
17. Benca, R. M., Obermeyer, W. H., Thisted, R. A., & Gillin, J. C. (1992). Sleep and psychiatric disorders. A meta-analysis. *Arch Gen Psychiatry*, *49*(8), 651-668; discussion 669-670. <https://doi.org/10.1001/archpsyc.1992.01820080059010>
